# QTL-seq reveals a major root-knot nematode resistance locus on chromosome 11 in rice (*Oryza sativa* L.)

**DOI:** 10.1007/s10681-019-2427-0

**Published:** 2019-06-14

**Authors:** Zobaida Lahari, Antonio Ribeiro, Partha Talukdar, Brennan Martin, Zeynab Heidari, Godelieve Gheysen, Adam H. Price, Roshi Shrestha

**Affiliations:** 1 Department of Biotechnology, Faculty of Bioscience Engineering, Ghent University, Ghent, Belgium; 2 Institute of Biological and Environmental Science, University of Aberdeen, Aberdeen, UK; 3 Centre for Genome-Enabled Biology and Medicine, University of Aberdeen, Aberdeen, UK

**Keywords:** *M. graminicola*, *O. sativa*, QTL-seq, Bulk segregant analysis, Nematode resistance genes

## Abstract

The root-knot nematode *Meloidogyne graminicola* is a serious pest in rice affecting production in many rice growing areas. Natural host resistance is an attractive control strategy because the speed of the parasite’s life cycle and the broad host range it attacks make other control measures challenging. Although resistance has been found in the domesticated African rice *Oryza glaberrima* and the wild rice species *O. longistaminata,* the introgression of resistance genes to Asian rice *O. sativa* is challenging. Resistance due to a major gene in *O. sativa* would greatly aid breeding. Recently two accessions resistant to *M. graminicola* have been identified in a screen of 332 diverse *O. sativa* cultivars. In this study, these two resistant cultivars, LD 24 (an *indica* from Sri Lanka) and Khao Pahk Maw (an *aus* from Thailand), were crossed with a moderately susceptible cultivar, Vialone Nano (a *temperate japonica* from Italy). Approximately 175 F_2_ progeny of both populations were screened for susceptibility to *M. graminicola* infection. Between 20 and 23 individuals with highest and lowest galls per plants were pooled to make susceptible and resistant bulks which were sequenced to conduct bulked segregant analysis using the QTL-seq method. This revealed a nematode resistance locus from 23 Mbp to the bottom of rice chromosome 11 in both crosses suggesting a rare introgression of the same locus is responsible for resistance in both cultivars. While this information can be used in marker-assisted breeding, analysis of available SNP data revealed candidate loci and genes worthy of further investigation for gene identification.

**Electronic supplementary material:**

The online version of this article (10.1007/s10681-019-2427-0) contains supplementary material, which is available to authorized users.

## Introduction

Rice (*Oryza sativa*) is an essential food crop hosting various pests and diseases including plant-parasitic nematodes which pose a threat to production. With over 41,000 species of plant-parasitic nematodes described (Decraemer and Hunt 2006), they pose a great threat to world agriculture. It has been estimated that plant nematodes alone can cause damage of around USD80 billion per year world-wide (Nicol et al. 2011).

Amongst plant parasitic nematodes, root-knot nematodes (RKN) are obligate parasites which are distributed all over the world with 98 different species infecting almost every plant species (Moens et al. 2009). The *Meloidogyne* genus was listed first in the top 10 most important plant pathogenic nematodes in a survey of 1100 members of the Nematology Society (Jones et al. 2013).

Within the genus *Meloidogyne*, the rice root-knot nematode (*M. graminicola)* (Golden and Birchfield 1965) is considered a serious threat to rice production (Plowright and Bridge 1990). The second stage juveniles (J2s) are the only infective stage of these nematodes and they invade rice roots near the root tip (Bridge et al. 2005). After migration into the stele, the J2s establish a feeding site consisting of giant cells in the vascular tissue. The infection causes the development of hook-like galls inside which the nematodes complete their life cycle (Mantelin et al. 2017).

*M. graminicola* damages upland, lowland, deep-water and irrigated rice (Bridge et al. 2005; Win et al. 2011) and yield losses of up to 80% have been reported (Padhgham et al. 2004; Soriano et al. 2000). Once inside the roots, they can multiply even under flooded conditions because the J2s hatch from an egg mass that is retained within the root in contrast to other RKN. The J2s might not be able to infect new roots under flooded conditions, but they can move to penetrate other plants as soon as the fields are drained. As water is getting scarce everywhere, water-saving rice production is being encouraged. This will make soil conditions more favourable for high *M. graminicola* reproduction (De Waele and Elsen 2007). To quote these authors “observations increasingly indicate that the large-scale introduction of these [water saving] techniques is favouring the development of high populations of *M. graminicola*, drastically increasing its economic significance”.

*M. graminicola* is widely distributed in many rice growing areas in South and Southeast Asia (Jain et al. 2012). Although *M. graminicola* is considered a serious pest in the tropics (Jones et al. 2013), it has recently been detected in rice fields in Italy (Fanelli et al. 2017). This is the first report of this pest in temperate rice production. This observation is important for two reasons. First, it fits with the prediction that major tropical pests will move north with global warming (Bebber et al. 2013) meaning breeders will have to incorporate new breeding targets. Second, when screening a global diversity panel Dimkpa et al. (2016) found temperate rice cultivars on average more susceptible to *M. graminicola*, presumably as resistance has not previously been selected for (deliberately or otherwise).

With the advent of plant molecular genetics, many nematode resistance genes or quantitative trait loci (QTLs) for resistance to plant nematodes have been mapped to chromosomal locations and some genes have been cloned. *Mi*-*1.2* is one of the best characterised root-knot nematode resistance genes which was found in a wild relative of tomato and confers resistance to several *Meloidogyne* species (Veremis and Roberts 2000). Similarly, *Hs1*^*pro*−*1*^, from wild beet against *Heterodera schachtii* (Cai et al. 1997), and *Gpa*-*2*, from potato against *Globodera pallida* (van der Vossen et al. 2000), are some of the identified natural resistance genes that can be used for developing nematode resistant cultivars. With the RKN infecting almost all the cultivars of *O. sativa* rice, it has been assumed that there is only a limited opportunity for breeding for nematode resistance using *O. sativa*. Natural resistance to *M. graminicola* has been reported in *Oryza longistaminata* and *Oryza glaberrima* (Soriano et al. 1999). However, *O. glaberrima*, the domesticated rice originating from West Africa, is low yielding and of minor economic importance compared to Asian rice *O. sativa* (Linares 2002). Introgression of *O. glaberrima* into *O. sativa* has lead, for example, to the new rice for Africa (NERICA) cultivars (Jones et al. 1997) but introgression of *M. graminicola* resistance from *O. glaberrima* to *O. sativa* has not been successful (Cabasan et al. 2017). Therefore natural resistance in *O. sativa* cultivars is potentially very important. In Asian rice, QTLs for partial resistance to *M. graminicola* have been reported on chromosomes 1, 2, 6, 7, 9, and 11 using the Bala × Azucena mapping population (Shrestha et al. 2007). Mapping of a *M. graminicola* resistance on chromosome 10 in Asian rice (cv. Abhishek) using bulk segregant analysis was reported by Mhatre et al. (2017). More recently, Phan et al. (2018) reported a Hypersensitivity-Like Reaction (HR) to *M. graminicola* infection in the Asian rice cultivar Zhonghua 11 suggesting this resistance to *M. graminicola* to be qualitative rather than quantitative involving (a) major gene(s). Galeng-Lawilao et al. (2018) have reported main effect QTLs for field resistance in Asian rice on chromosomes 4, 7 and 9 plus two epistatic interactions (between loci on chromosome 3 and 11, and between 4 and 8).

Screening of 332 cultivars of a global rice panel, Rice Diversity Panel 1 (RDP1) identified two Asian rice cultivars, LD 24 and Khao Pahk Maw (KPM) to be resistant to *M. graminicola* (Dimkpa et al. 2016). In that study, data on 44,000 SNP markers was used to suggest three loci where a resistance locus might reside (around 42 MbP on chromosome 1, 1 Mbp on chromosome 3 and 26 Mbp on chromosome 11) assuming that resistance in both cultivars was the result of the introgression of rare alleles of the same major resistance gene.

Bulk segregant analysis (BSA) has proved to be an effective way to locate genes or QTLs from populations with two extreme phenotypic traits, which is most applicable to segregation of major genes (Michelmore et al. 1991; Trick et al. 2012; Venuprasad et al. 2009). In the past, BSA has been an important tool for rapidly identifying markers in a genomic region associated with a trait of interest (Giovannoni et al. 1991). QTL-seq is a novel and rapid way for performing bulk segregant analysis using next-generation sequencing data which was first reported by Takagi et al. (2013) who used it to identify QTLs for partial resistance to rice blast. In principle, this calculates a SNP index as the relative frequency of the parental alleles for each SNP in both the resistant and susceptible pools, then calculates a delta-SNP index as the difference between the proportions from the two bulks. Regions with a delta SNP-index that pass a confidence interval threshold, as calculated by statistical simulation, should contain a QTL. This method has recently been used to detect major QTLs in several crops (Illa-Berenguer et al. 2015; Nowak et al. 2015; Sagawa et al. 2016; Shu et al. 2018).

Two cultivars (LD 24 and Khao Pahk Maw) identified as resistant to *M. graminicola* (Dimkpa et al. 2016) were crossed with a susceptible cultivar (Vialone Nano). The main objective of this current study was to use the QTL-seq method to test the hypothesis that these two resistant cultivars which are themselves genetically quite different, harbour the same allele for resistance, and identify loci and candidate genes for conferring resistance to *M. graminicola* with the long-term goal of improving nematode resistance in cultivated rice, *O. sativa*.

## Methods

### Plant materials and screening for nematode gall formation

Two recombinant F_2_ populations were used to screen for *M. graminicola* resistance. The first population (LD 24 × VN) was the progeny from the nematode resistant LD 24, which is an *indica* from Sri Lanka, crossed with the susceptible Italian *temperate japonica* cultivar, Vialone Nano (VN). For the other population (VN × KPM) Vialone Nano was crossed with the resistant *aus* rice cultivar Khao Pahk Maw (KPM) from Thailand. LD 24 and KPM are part of the Rice Diversity Panel 1 (Zhao et al. 2010) and seeds were originally obtained from the National Rice Research Centre, USA, and bulked in Aberdeen, UK. Seeds of VN were obtained from Giampiero Vale of the Consiglio per la Ricerca e la Sperimentazione in Agricoltura (CRA), Vercelli, Italy.

The screenings were carried out at Ghent University, Belgium using 178 individual F_2_ plants of LD 24 × VN and 174 individual F_2_ plants of VN × KPM. Seeds were first pre-germinated in petri dishes at 30 °C for 4 days in dark. Each germinated seedling was planted into a specially made polyvinylchloride tube containing sand and absorbent polymer (Reverstat et al. 1999). Then the seedlings were grown in a rice culture room under controlled environmental conditions (26/24 °C day/night temperature, 70% relative humidity, 12/12 h light/dark cycle). Each plant was fertilized with 10 ml of Hoagland’s solution 2 times per week. The root-knot nematode, *M. graminicola* was provided by Prof. Dirk De Waele (University of Leuven, Belgium) and was originally isolated from rice in Philippines. They were multiplied and maintained using a susceptible rice genotype Nipponbare or the grass host *Echinocloa crusgalli*. The second stage juveniles (J2) of *M. graminicola* were extracted from 2 to 3 months old infected roots using the modified Baermann method and 200 J2s per plant was added to 2-week old seedlings. Two weeks after inoculation, the plants were individually washed and stained with acid fuchsin (Byrd et al. 1983) to count the number of galls per plant.

For the LD 24 × VN cross, six separate batches of screening were conducted assessing 26–34 F_2_ plants with check cultivars of the parents and Nipponbare. For the VN × KPM cross, five separate batches of screens were conducted assessing 33–37 F_2_ plants with checks of the parents and Nipponbare. The results are shown as histograms for each screening run in Online Resources 1 and 2.

The resistant pool for the first population (LD 24 × VN) was made from 23 individual plants with no or few galls (0–2 galls) and the susceptible pool was made from 23 individuals with higher gall numbers per plant (10–34 galls). Similarly, the resistant pool of the second population (VN × KPM) was made from 20 plants with low numbers of galls per plant (0–4 galls) and the susceptible bulk contained 20 individual plants with a high number of galls per plant (21–47 galls). The CTAB method (Murray and Thompson 1980) was used to extract DNA from the bulks and from 10 or 11 individual plants of each of the parents LD 24, KPM and VN.

### Applying QTL-seq

The DNA from pooled bulks and parent samples were quantified using the Thermo Fisher Scientific Qubit dsDNA BR Assay Kit (Thermo Fisher Scientific, Waltham, MA, USA) on the Thermo Fisher Scientific Qubit 2.0 Fluorometer. The quality and size of the DNA was analysed on an Agilent 2200 TapeStation (Agilent Technologies, Santa Clara, CA, USA) with the gDNA ScreenTape. Fragmentation of the gDNA prior to library preparation was performed with the Bioruptor Pico sonication device from Diagenode (Diagenode SA, Seraing, Belgium). The fragmented DNA samples were prepared for sequencing and barcoded using the TruSeq DNA Nano Library Preparation kit (Illumina Inc., San Diego, CA, USA). The libraries were quantified by qPCR with the KAPA Complete for Illumina Library Quantification Kit (Roche Diagnostics, Risch-Rotkreuz, Switzerland) on a Thermo Fisher Scientific QuantStudio 6 Flex Real-Time PCR System. The libraries were analysed for size and quality on an Agilent 2200 TapeStation using D1000 and D5000 ScreenTapes. The resultant barcoded libraries were equimolar pooled and sequenced on an Illumina MiSeq Sequencing System using MiSeq v3 chemistry with 300 bp paired-end reads. Base calling and FASTQ output files were generated on the MiSeq instrument. The total reads obtained are given in Table 1. On average, each sequence yielded about 10 × coverage of the rice genome. Sequence data is available in the European Nucleotide Archive (EBI-ENA) with primary accession number PRJEB27629.

**Table 1:** Quantity of genome sequence obtained for each sample

Sample	Base pair reads (bp)
LD 24 × VN resistant (R) pool	3,425,008,208
LD 24 × VN susceptible (S) pool	3,535,058,903
VN × KPM resistant (R) pool	4,797,233,723
VN × KPM susceptible (S) pool	4,132,402,603
LD 24 parent pool	3,947,278,012
KPM parent pool	4,849,506,583
VN parent pool	3,789,900,665

Quality assessment of read data was performed for all samples using FASTQC (version 0.11.5; Andrews 2010) and MultiQC (version 1.1; Ewels et al. 2016) using default parameters. Raw reads from each of the samples were filtered to remove poor quality sequences and trimmed to remove contaminating adapter sequences as well as any unwanted bias from their ends using Trim Galore! (Version 0.4.0; Krueger 2012). A Phred score of 30 was used as the overall quality threshold for the tool.

### Complementary SNP calling

All samples were subjected to complementary independent SNP calling by having, firstly, their corresponding read datasets aligned to the ENSEMBL’s release 32 Oryza_sativa.IRGSP-1.0 reference sequence of cultivar Nipponbare using BWA-MEM algorithm (version 0.7.12-r1039; Li and Durbin 2010). Alignments obtained were, respectively, sorted and had duplicates marked with Samtools (version 0.1.19-44428 cd; Li et al. 2009) and Picard (version 1.104; http://broadinstitute.github.io/picard). All tools were configured with default parameters. Subsequently, FreeBayes (version v0.9.14; Garrison and Marth 2012) was used to perform the SNP calling task over each alignment file using parameters –m 20 –q 20 –n 4 –J –j –min-repeat-entropy 1 –no-partial-observations –F 0.1 –C 2. Ploidy parameter –p was adjusted according to the number of individuals in each sample.

Two instances of the QTL-seq pipeline (version 1.4.4; Takagi et al. 2013) were employed to analyse the SNP profiles of the respective S- and R-bulks of both populations. In the first one, the S-bulk from the cross LD 24 × VN was set up as bulk “A” while the R-bulk was set up as the “B” one. Similarly, in the second instance, the S-bulk from the cross VN × KPM was set up as bulk “A” while the R-bulk was set up as bulk “B”. Respectively, the genotypes of both parents LD 24 and KPM were used to develop the reference sequences. In both scenarios, the ENSEMBL’s release 32 Oryza_sativa.IRGSP-1.0 FASTA file was used as the public genome sequence. SNP-index and Δ (SNP-index) were calculated to identify the region of interest and plotted on chromosome maps (Takagi et al. 2013). Each Δ (SNP-index) was obtained by subtracting the respective SNP-index value of the R-bulk from the SNP-index value of the S-bulk. Due to the previous quality control step of the reads, the “Qualify read” stage of the QTL-seq pipeline was configured with Phred score values of 20.

## Results

### Phenotype

**Fig. 1:**
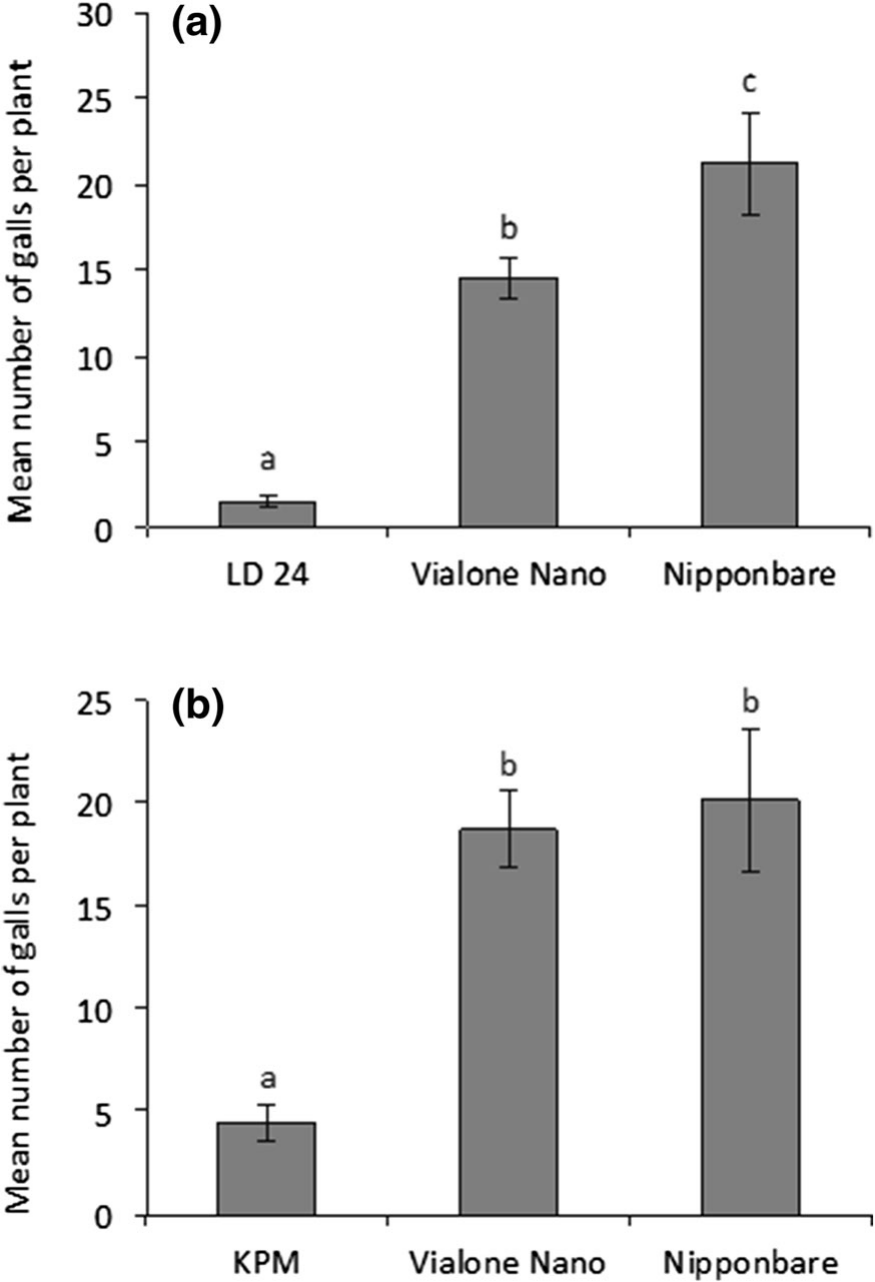
Mean number of galls per plant in the parents (LD 24, KPM and VN) of the two crosses and the check variety, Nipponbare. Two week old seedlings were inoculated with 200 J2s and the number of galls per plant were assessed after another 2 weeks. **a** Data for LD 24 and VN are means of 11 seedlings from five screens, and Nipponbare 10 seedlings from four screens. **b** Data for KPM and VN are means of 10 seedlings from five screens, and Nipponbare 9 seedlings from four screens. Data were analyzed by one-way ANOVA followed by Tukey’s post hoc test. Different letters indicate means were statistically different at 95% confidence. Bar = standard error

**Fig. 2:**
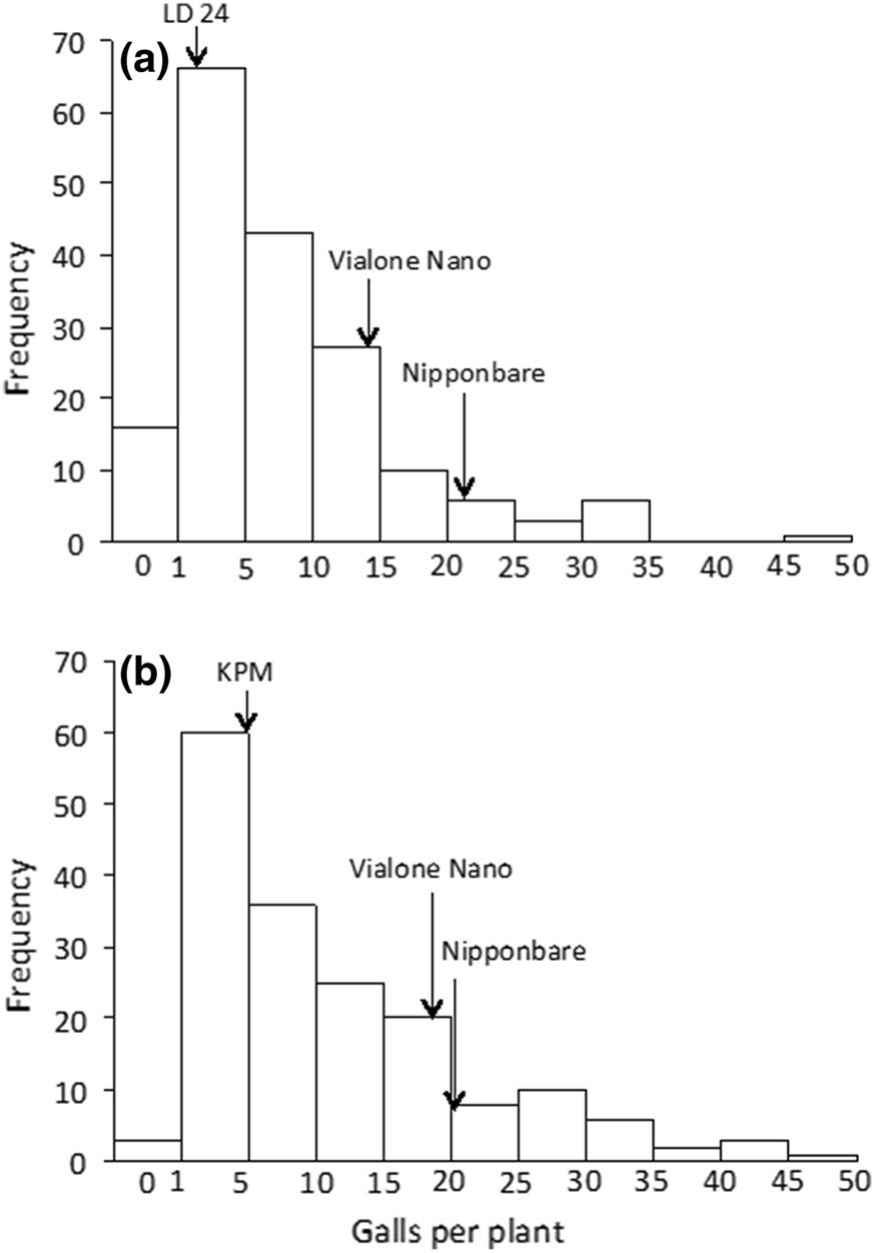
Frequency distribution of galls per plant in the two populations **a** LD 24 × VN and **b** VN × KPM. In **a** n = 178 for F_2_, 11 for LD 24, 11 for VN and 10 for Nipponbare. In **b** n = 174 for F_2_, 10 for KPM, 10 for VN and 9 for Nipponbare

LD 24 and KPM are two *O. sativa* cultivars that were previously identified as resistant to *M. graminicola* in assessments conducted in the authors’ labs in both Aberdeen and Ghent (Dimkpa et al. 2016). The resistance was confirmed here with the numbers of galls per plant being significantly lower in those two genotypes compared to Nipponbare or the other parent of the crosses (Vialone Nano) (Figs. 1, 2). Vialone Nano can be considered as susceptible since it performed similarly to the susceptible Nipponbare (Fig. 1) and Nipponbare was found to be moderately susceptible by Dimkpa et al. (2016). In every batch the F_2_ plants showed a wide distribution of gall numbers from as low as the resistant parent to higher than the susceptible cultivars (Online Resources 1 and 2) indicating genetic segregation suitable for BSA.

**Fig. 3:**
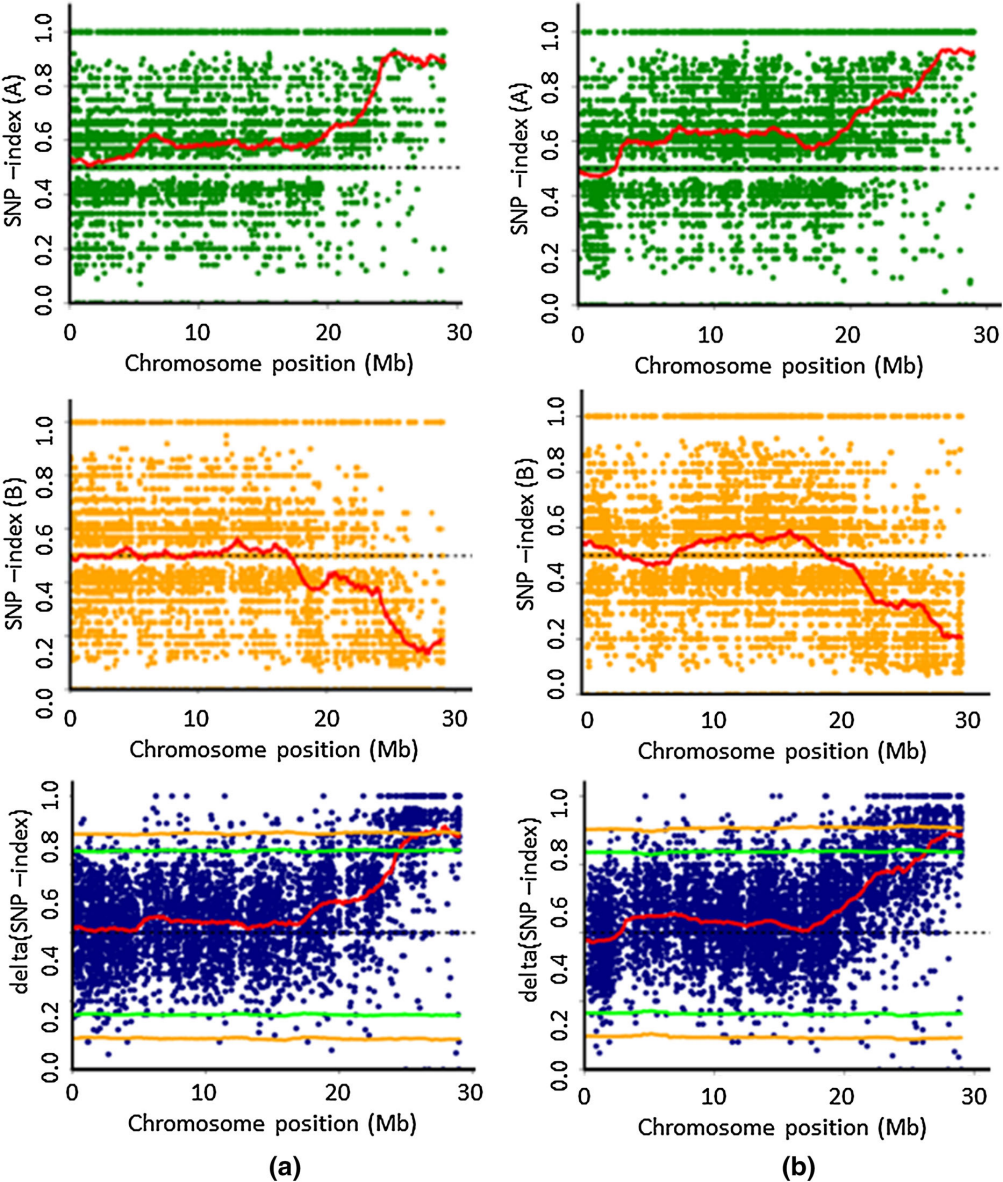
Single nucleotide polymorphism (SNP)-index charts of S-bulk (green) and R-bulk (orange) and corresponding Δ(SNP-index) plots (green) of chromosome 11 with 95–99% confidence interval borders of **a** LD 24 × VN and **b** VN × KPM. Average values of Δ (SNP-index) plotted with a 2 Mb sliding window and a 50 kb increment. Results of QTL-seq for all chromosomes, in both crosses, are shown in the Online Resources. (Color figure online)

QTL-seq is a tool for assessing allele frequency in bulked DNA samples. In such analysis, the SNP-index represents the frequency of the allele that is different from one of the cultivars used to develop the reference sequence. Thus, the SNP-index pattern is dependent on the developed reference sequence. For example, if LD 24 was used to develop the reference sequence, the SNP-index in the genomic region where the bulked DNA has a high frequency of the same allele of LD 24 should be close to 0. Conversely, the genomic region where the bulked DNA has a high frequency of the allele that is different to LD 24 should show a SNP-index close to 1. The susceptible bulk DNA, thus, should contain low frequency of the LD 24 allele and high frequency of Vialone Nano allele. Conversely, the resistant bulk DNA will contain low frequency of Vialone Nano allele and high frequency of the LD 24 allele (Fig. 3a). The Δ (SNP-index) was calculated for each SNP between the bulks using sliding window analysis. The Δ (SNP-index) values were plotted onto the chromosome with 95-99% confidence interval (CI) (or statistical confidence *p *< 0.01 and < 0.05; Fig. 3). The orange line indicates 99% CI whereas the green line indicates 95% CI. The red line is the sliding window average of the Δ (SNP-index). Online Resources 3 and 4 show the Δ (SNP-index) values plotted for all chromosomes in each cross respectively. In both the LD 24 × VN and the VN × KPM analysis a distortion of segregation as revealed by significant delta SNP index appears only on chromosome 11. Figure 3 shows the SNP index for resistant and susceptible bulks plus the delta SNP index for chromosome 11 for both crosses. It shows the presence of a QTL at the bottom of chromosome 11 from 23 Mbp and down (to the end of the chromosome at 29 Mbp).

### SNP analysis on the bottom of chromosome 11

**Fig. 4:**
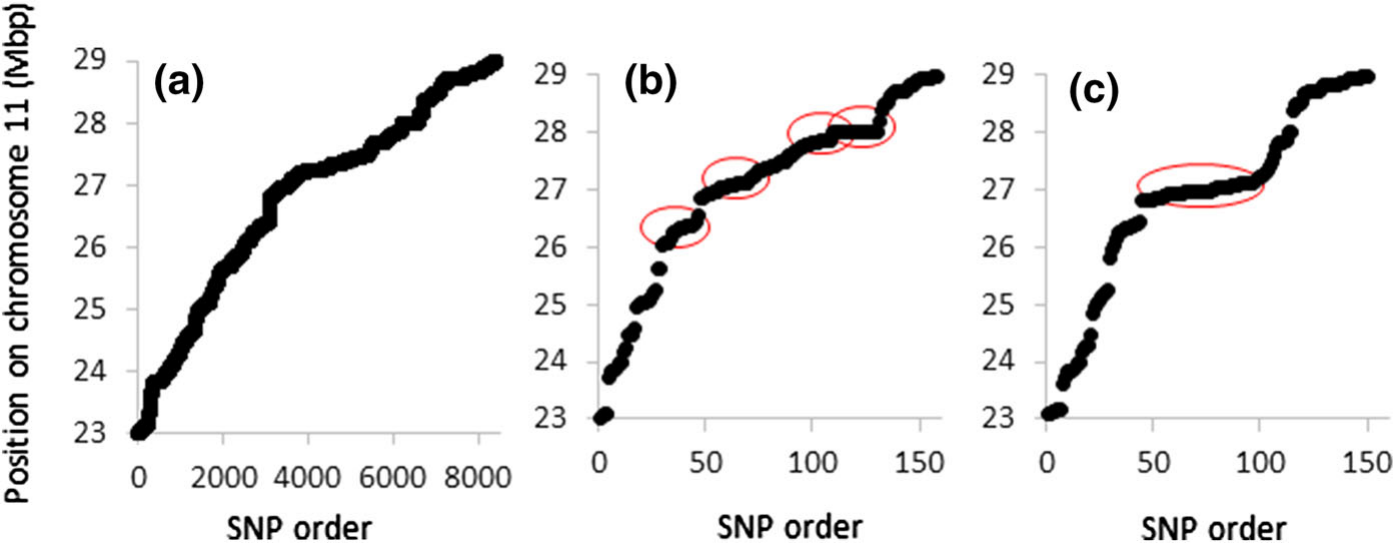
Distribution of SNPs by their order on the chromosome (from the 23 Mbp) (x axis) and their exact position on the chromosome (y axis) for **a** 8440 SNPs common to LD 24, KPM and resistant bulks but different to VN and susceptible bulks, **b** subset of the 8440 which are different between KPM and gall-supporting cultivar Seratoes Hari which is genetically similar using 44 k SNPs and **c** subset of the 8440 which are also different between KPM and gall-supporting cultivars 27, ARC 10086 and PTB 30 which are genetically similar to KPM using 12,268 SNPs from 23 Mbp to the end of chromosome 11. Circled in red are regions where SNPs appear to cluster suggestive of an introgressed region responsible for resistance. (Color figure online)

An analysis of polymorphisms (SNPs and small indels) detected in the sequencing conducted here and available data on SNPs in the High Density Rice Array (HDRA) were used to search for the most likely location of the locus underlying resistance, focusing on chromosome 11 from 23 Mbp to the end (29 Mbp). If the resistance in LD 24 and KPM is the result of the introgression of a resistance gene from outside *O. sativa*, there should be a region of genomic sequence shared between LD 24 and KPM that contains the gene(s) responsible for resistance which is (1) not present in either VN or the Nipponbare reference, and (2) rare within *O. sativa*. The available SNP data (obtained here but also available elsewhere) should give some clue as to the location of that locus as a cluster of LD 24 and KPM specific SNPs would be expected around the locus, the size of the cluster being dependent on the size of the introgression that has introduced the resistance. There were 8440 polymorphisms which fulfilled the following criteria: one allele was common between LD 24, KPM, the LD 24 × VN resistant bulk, and the VN × KPM resistant bulk, while the other allele was common between VN and the susceptible bulks. A total of 5500 of these were null alleles in the resistant genotypes (the SNP was absent in the resistant genotypes). These polymorphisms were distributed evenly over the 6 Mbp region (Fig. 4a). Two further steps were used to filter polymorphisms using the HDRA data of 700 k SNPs (McCouch et al. 2016) available on the IRRI SNP-Seek database. All SNPs for the HDRA, from 23 Mbp to the end of chromosome 11, were downloaded. Unfortunately, LD 24 data is not available despite being available for the 44 k data set (Dimkpa et al. 2016). Using principle component analysis on the 44 k SNP dataset the cultivar Seratoes Hari was identified by Dimkpa et al. (2016) to be genetically very similar to LD 24, but in contrast being susceptible to *M. graminicola* infection. We therefore listed all the SNPs detected above which were not common with Seratoes Hari. Only 159 SNPs meet this criterion and they are not evenly distributed over the 6 Mbp region (Fig. 4b). There is some evidence of clustering around 25.1, 26.4, 27.8, and 28.0 Mbp. A further analysis was conducted considering that, when comparing similarity between genotypes tested for galling reported in Dimkpa et al. (2016), rather than using 44 K SNPs distributed across the genome, it would be more appropriate here to use only SNPs from 23 Mbp to the end of chromosome 11. This amounts to 12,268 SNPs. Tassel (V5) (Bradbury et al. 2007) was used to calculate a distance matrix on just the RDP1 genotypes, revealing cultivar 27 (NSFTVID 242; *tropical japonica*), ARC 10086 (NSFTVID 358; *temperate japonica*) and PTB 30 (NSFTVID 360; *aus*) are all very similar to KPM at the bottom of chromosome 11 but support nematode infection. Comparing SNPs in these cultivars with KPM revealed 1770 SNPs where KPM is different. These are distributed evenly over the 6 Mbp region. Examining which of these SNPs are also in the 8440 SNPs that are common between KPM and LD 24 from our data reveals 151 SNPs and these are clustered around 26.9 Mbp (Fig. 4c).

## Discussion

QTL-seq was used to identify the genomic region involved in resistance to the rice root-knot nematode (*M. graminicola*) in *O. sativa* cultivars LD24 and KPM. Bulked segregant analysis (BSA) is a mapping technique used to identify DNA markers linked to a particular locus. In the current study, paired bulked DNA samples were developed from two populations segregating for nematode susceptibility in terms of number of galls per plant. Resistant and susceptible bulks were generated by pooling DNA from plants with low gall numbers and plants with high gall numbers respectively.

The success of bulk segregant analysis depends on the heritability of the QTL in question. This is maximised if the QTL has a large effect or even more so if there is a single major gene responsible. It is also maximised by using a large population and having low error in the estimate of phenotype. Here we use a large population (approximately 175) but phenotyping was performed on an F_2_ which does not allow replication. The trait used here tends to have quite high variation between replicates (e.g. coefficient of variance tended to range from 50 to 100% in screen of RDP1 as reported in Dimkpa et al. (2016)). This means that the approach used here was only likely to work if great care was taken in phenotyping and if the variation was explained by a major QTL or major gene. The fact that the approach worked, revealing loci on the bottom of chromosome 11 in both crosses, validates the decision to progress rapidly without generating F_3_ material that would have allowed replicated phenotyping.

Both QTL-seq experiments revealed segregation from 23 Mb to the bottom of chromosome 11. This locus does not seem to be close to previously detected QTLs for nematode resistance on chromosome 11 from Shrestha et al. (2007) or Galeng-Lawilao et al. (2018). Within this region there are 859 annotated genes (according to the Rice Genome Annotation Project) including 88 (annotated as) transposons and 167 retrotransposons, 231 “expressed protein” and 29 hypothetical genes. Within this list there are 30 NBS containing disease resistance genes, eight other “disease resistance protein” genes, three “stripe rust resistance protein” genes, two “rust resistance protein” genes, two *RGH* genes (also resistance genes) and *MLA10*, a mildew resistance gene. This region on chromosome 11 corresponds to one identified by Dimkpa et al. (2016) as a potential location for a nematode resistance gene. Dimkpa et al. (2016) used 44 k SNP data to reveal 16 SNPs that were common between KPM and LD 24, not shared with the cultivar Seratoes Hari (a susceptible cultivar closely related to the resistant LD 24) and which are rare in the Rice Diversity Panel 1. Three of these are at 26.3 Mbp on chromosome 11, within the region containing the QTL detected here. Importantly, the authors acknowledged that the approach assumed that the resistance locus was the same between the two resistant cultivars. This assumption seems to be valid since both crosses reveal the same locus with QTL-seq. A similar approach to that of Dimkpa et al. (2016) was used to try to narrow down the likely position of the resistance genes using the expanded SNP data set provided by sequencing reported here. A total of 8440 SNPs in the region from 23 to 29 Mbs on chromosome 11 were common between LD 24 and KPM and both resistant bulks, but different to VN and the susceptible bulks. Since these are spread over the region, and not clustered (Fig. 4a) it does not help to predict the locus more accurately. The RDP1 has been assessed with a 700 K array (McCouch et al. 2016) meaning it is possible to integrate that data with the SNPs detected by QTL-seq. Only 159 SNPs occur in both the 8440 revealed by QTL-seq and the 700 K SNP database and are different to Seratoes Hari. These predominantly fall into four clusters at 25.1, 26.4, 27.8, and 28.0 Mbp (Fig. 4b). Rather than the global SNP analysis that identified Seratoes Hari reported by Dimkpa et al. (2016), a slightly different approach was used to exploit the 700 K SNP data available for the Rice Diversity Panel. This allowed the identification of three cultivars susceptible to *M. graminicola* but none the less very similar to KPM only in this region (23–29 Mbp of chromosome 11 only). There were 1770 SNPs which differ between these cultivars and KPM from this part of chromosome 11. Only 151 of these SNPs also differentiate KPM, LD 24 and the resistant bulks from VN and the susceptible bulks and these are clustered around 26.9 Mbp (Fig. 4c).

**Fig. 5:**
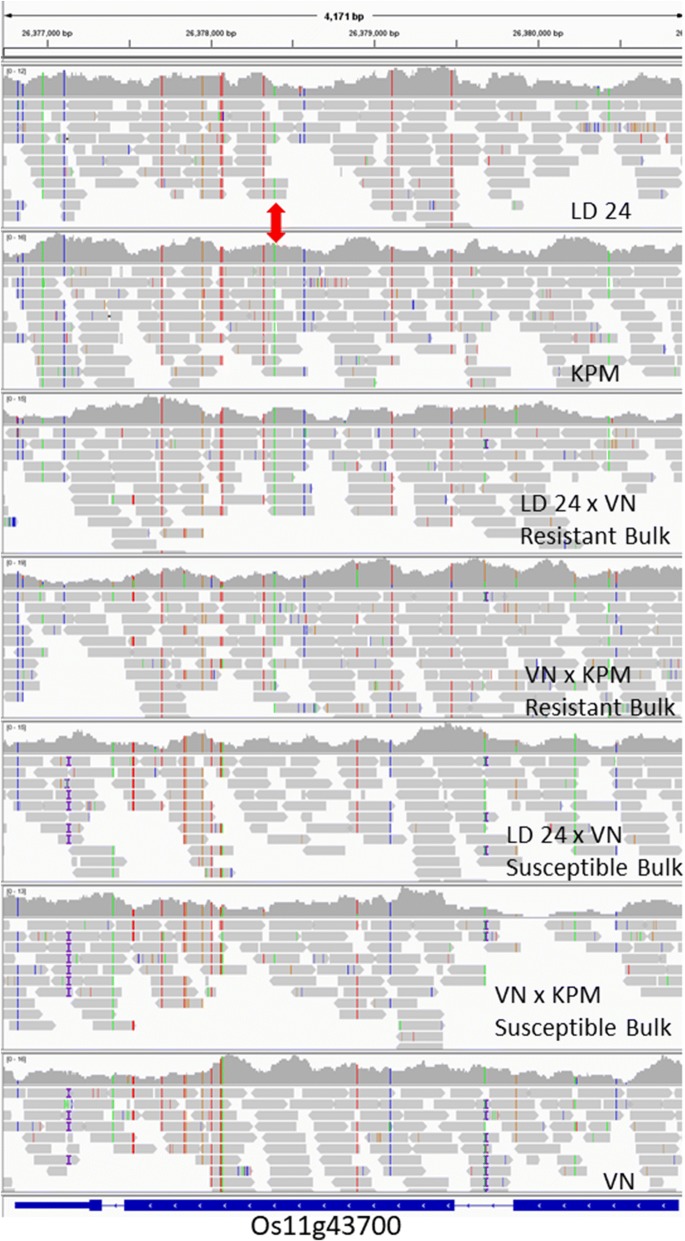
Screen dump of Integrative Genome Viewer showing LOC_Os11g43700 *RGH1A* candidate gene in the resistant parents, resistant bulks, susceptible bulks and susceptible parents. One SNP, 26,378,391 is highlighted with the red arrow as it is absent in RDP1 cultivars Seratoes Hari, 27, ARC10086 and PTB30

The resistance gene homolog (LOC_Os11g43700) annotated as *RGH1A* that was highlighted by Dimkpa et al. (2016) at 26.4 Mbp is at one of the clusters revealed by comparison with Seratoes Hari so must still be considered a good candidate gene. Indeed, examining the sequence reads within this gene for all sequences obtained here using the Integrative Genome Viewer (IGV) (Robinson et al. 2011), suggests it perfectly fits with LD 24 and KPM sharing an identical allele with the resistant bulks while VN and the susceptible bulks have a different allele (Fig. 5). Figure 5 shows many SNPs detected with respect to Nipponbare, several of which are specific either to just the resistant parents and resistant bulks, or to the susceptible parent and the susceptible bulks. In addition to SNPs, there is a 14 bp insertion in the intron and a 1 bp insertion in the 3′ UTR of VN and susceptible bulks. Importantly, LD 24 and KPM appear identical for this gene. One of these SNPs at 26,378,391 was not only common to the resistant parents and resistant bulks but also was not present in Seratoes Hari and three susceptible cultivars that are genetically very similar to KPM in this region. This SNP is non-synonymous replacing amino acid 705:arginine with a leucine. There are another six SNPs between LD24/KPM and Nipponbare in the predicted coding region of this gene, and unusually all but one are non-synonymous (aa 345, 468, 728, 812, 816). The gene has the coiled-coil, NB and LRR domains similar to *Gpa2*, the potato cyst nematode resistance gene (van der Vossen et al. 2000). Further investigation of this candidate gene seems warranted.

There are excellent candidate genes in the other clusters reported here. Notably, there is an NBS-LRR resistance gene (LOC_Os11g44580) at 26.95 Mbp while between 27.8 and 27.9 Mbp there are five NBS-LRR genes (LOC_Os11g45930, 45970, 45980, 46080 and 46100 two rust stripe resistance proteins (LOC_Os11g46130 and 46140) and an *MLA10* gene (LOC_Os11g46070). The *RGH1A*, rust stripe resistance and the *MLA* gene are all similar at the sequence level, with the *MLA* genes having been associated with nematode resistance (Wei et al. 2002). Importantly, from examination of sequence reads on IGV the genes listed above from LOC_Os11g46070 to 46140 all appear to be missing in LD 24 and KPM (see Online Resource 5 for IGV screen dump of 5 genes in this region). If this observation truly reflects the absence of these genes in LD 24 and KPM they cannot be candidate genes for resistance. It is possible that the genes missing here in LD 24 and KPM are disease susceptibility genes. This class of gene was introduced by Vogel (2002) and described genes required for susceptibility, and their molecular mechanism reviewed by Van Shie and Takken (2014). It must be noted, however, that both the alignment of QTL-seq reads and the listing of genes (above) is based on Nipponbare and its annotation. The strong possibility exists that the resistance locus discovered here represents sequence variation that is not present in Nipponbare rendering the alignment to Nipponbare problematic. If that is the case, de-novo assembly of KPM and LD 24 in this region should reveal the true nature of the genome relevant to the resistance locus, and that would require higher sequence depth and a diversity of sequencing methodologies to give some long reads. This may offer a method to identify the responsible gene(s) more rapidly than fine mapping and map-based cloning. In advance of that, this 6 Mbp region of chromosome 11 can be used for marker assisted selection of resistance to *M. graminicola*.

## Conclusion

This is the first report of bulk segregant analysis using QTL-seq to identify a nematode resistance locus in rice. Although the two resistant cultivars used in this study (LD 24 and KPM) are genetically different, the same locus on chromosome 11 was found to be responsible for *M. graminicola* resistance in both cultivars. Through the analysis of SNP data, we were able to identify some candidate genes that might confer resistance to *M. graminicola* in *O. sativa.* This locus can be used for marker-assisted breeding but further sequencing in the resistant parents and functional analysis of these candidate genes should facilitate gene identification for better biological understanding and improved resistance breeding.

## Electronic supplementary material

Below is the link to the electronic supplementary material.

**Online Resource 1** Figure of frequency distribution of galls per plant in each batch of LD 24 × VN cross (TIFF 82 kb)

**Online Resource 2** Figure of frequency distribution of galls per plant in each batch of VN x KPM cross (TIFF 76 kb)

**Online Resource 3** The Δ (SNP-index) charts obtained by subtraction of R-bulk SNP-index from S-bulk SNP-index for F_2_ obtained from a cross between LD 24 and VN, of all 12 chromosomes, with 95% and 99% confidence interval borders (TIFF 570 kb)

**Online Resource 4** The Δ (SNP-index) charts obtained by subtraction of R-bulk SNP-index from S-bulk SNP-index for F_2_ obtained from a cross between KPM and Vialone Nano, of all 12 chromosomes, with 95% and 99% confidence interval borders (TIFF 451 kb)

**Online Resource 5** Screen dump of Integrative Genome Viewer showing LOC_Os11g46100 to Os11g46140, part of a region apparently absent in the resistant parents and resistant bulks (TIFF 433 kb)
