# Supplementary figures and images for: QTL-seq reveals a major root-knot nematode resistance locus on chromosome 11 in rice (Oryza sativa L.)
Source: Euphytica. 2019 Jun 14;215(7):117. doi: 10.1007/s10681-019-2427-0 (PMC6570777; doi:10.1007/s10681-019-2427-0)

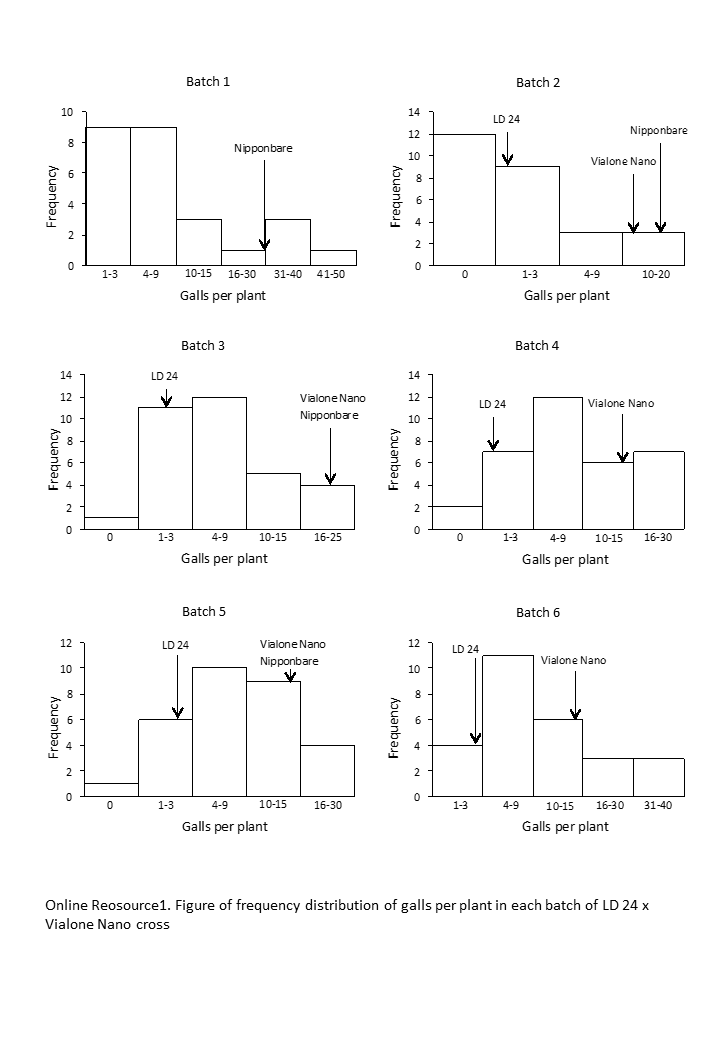

Supplement: Supplementary file 1 — Online Resource 1 Figure of frequency distribution of galls per plant in each batch of LD 24 × VN cross (TIFF 82 kb) [file 10681_2019_2427_MOESM1_ESM.tif]

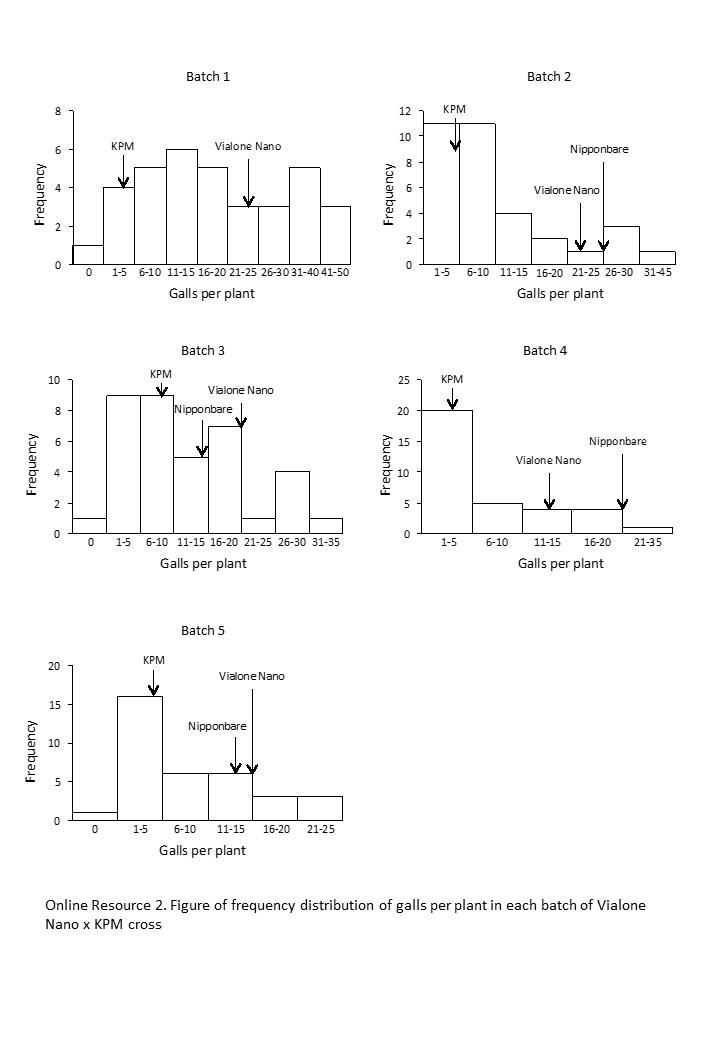

Supplement: Supplementary file 2 — Online Resource 2 Figure of frequency distribution of galls per plant in each batch of VN x KPM cross (TIFF 76 kb) [file 10681_2019_2427_MOESM2_ESM.tif]

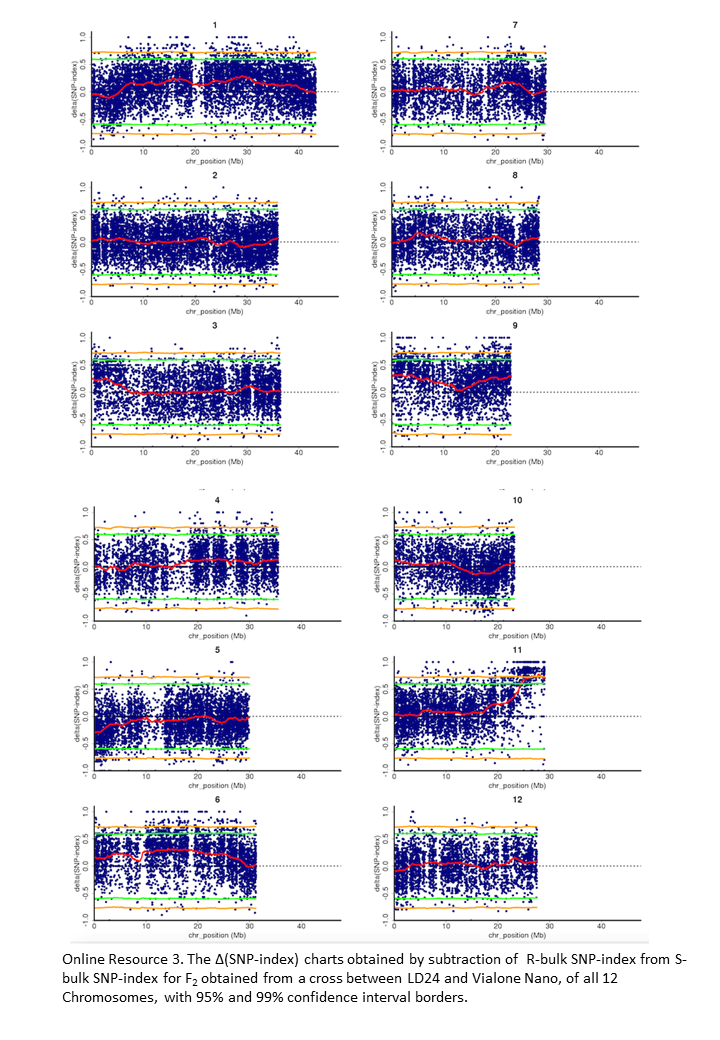

Supplement: Supplementary file 3 — Online Resource 3 The Δ (SNP-index) charts obtained by subtraction of R-bulk SNP-index from S-bulk SNP-index for F2 obtained from a cross between LD 24 and VN, of all 12 chromosomes, with 95% and 99% confidence interval borders (TIFF 570 kb) [file 10681_2019_2427_MOESM3_ESM.tif]

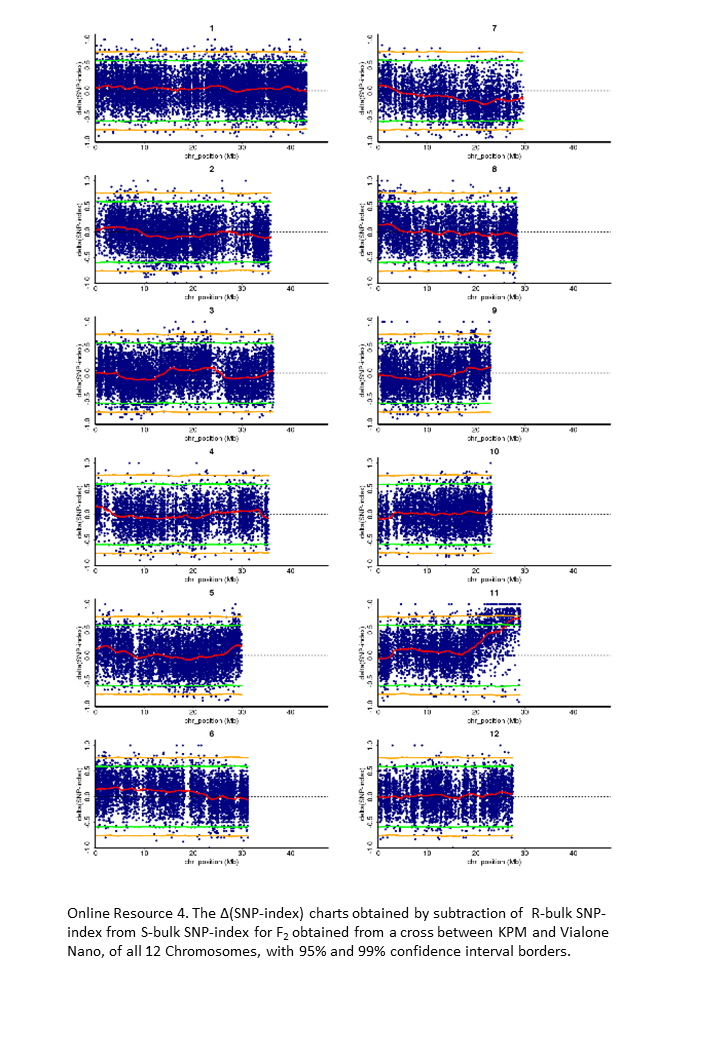

Supplement: Supplementary file 4 — Online Resource 4 The Δ (SNP-index) charts obtained by subtraction of R-bulk SNP-index from S-bulk SNP-index for F2 obtained from a cross between KPM and Vialone Nano, of all 12 chromosomes, with 95% and 99% confidence interval borders (TIFF 451 kb) [file 10681_2019_2427_MOESM4_ESM.tif]

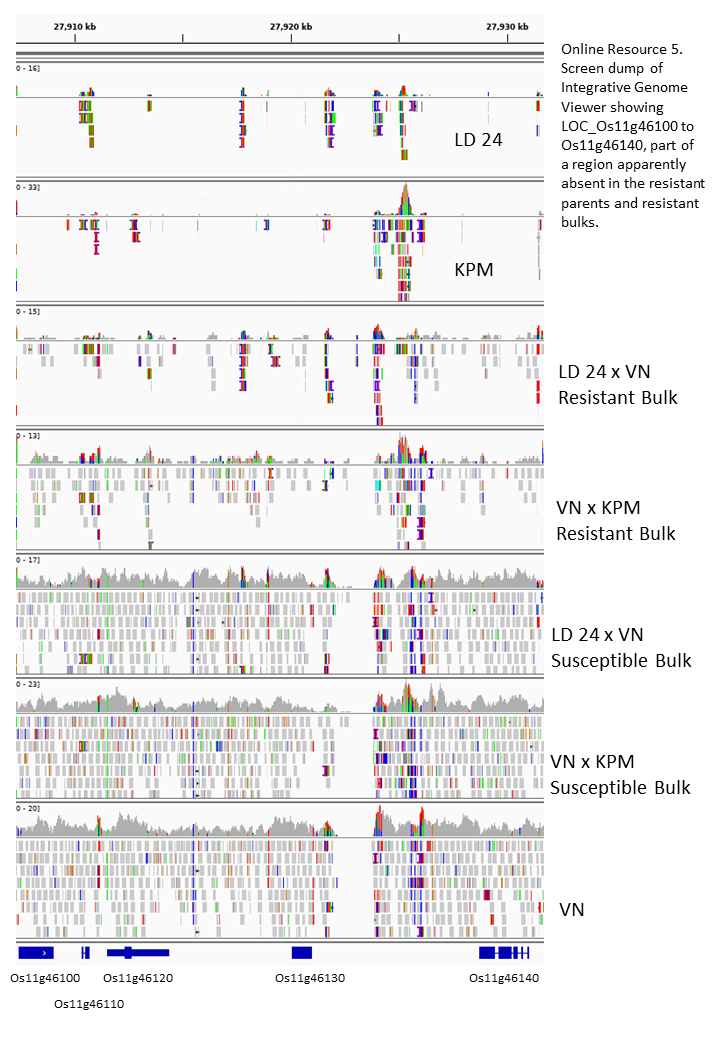

Supplement: Supplementary file 5 — Online Resource 5 Screen dump of Integrative Genome Viewer showing LOC_Os11g46100 to Os11g46140, part of a region apparently absent in the resistant parents and resistant bulks (TIFF 433 kb) [file 10681_2019_2427_MOESM5_ESM.tif]
